# Supplementary material for: Overexpression of the Transcription Factor GmbZIP60 Increases Salt and Drought Tolerance in Soybean (Glycine max)
Source: Int J Mol Sci. 2025 Apr 7;26(7):3455. doi: 10.3390/ijms26073455 (PMC11989446; doi:10.3390/ijms26073455)
Supplement: Supplementary file 1 [file ijms-26-03455-s001.zip › Supplemental Figures.pdf]

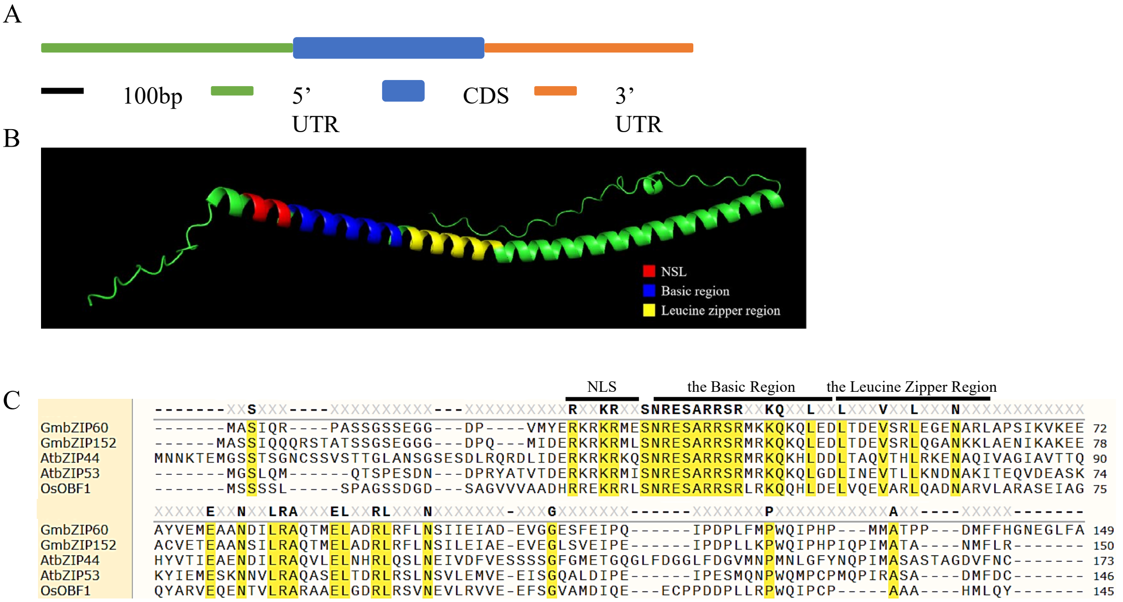

Supplemental Figure S1. Bioinformatics analysis of GmbZIP60. (A) The gene structure analysis. (B) The protein structure analysis. (C) Multiple alignments of the conserved basic region and the leucine zipper region between GmbZIP60, GmbZIP152, AtbZIP44, AtbZIP53, and OsOBF1.

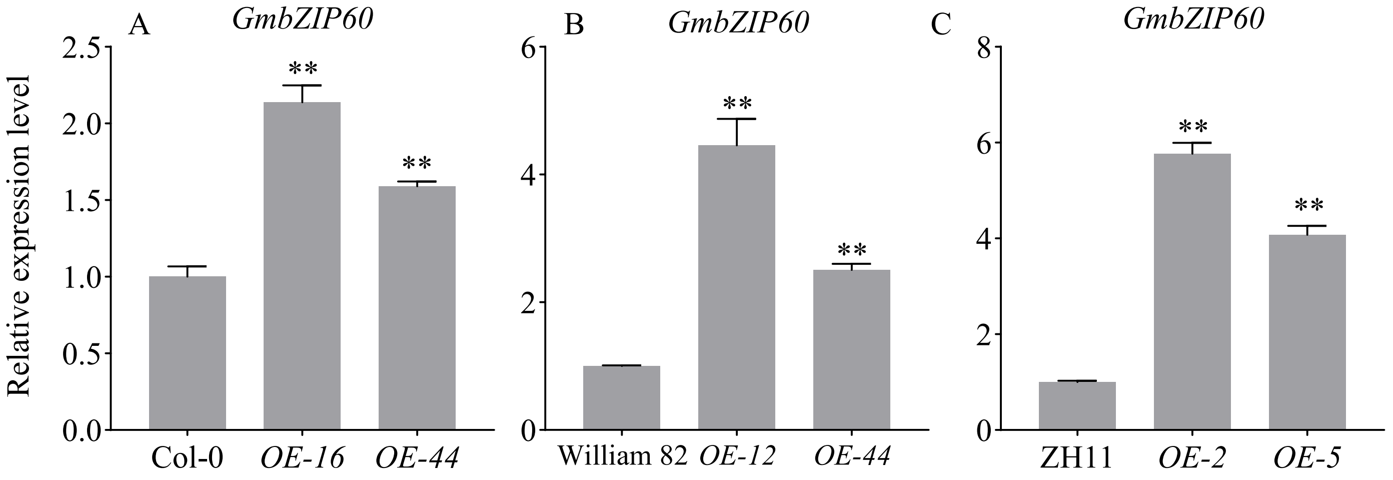

Supplemental Figure S2. Analysis of expression level of *GmbZIP60* in transgenic *Arabidopsis*, rice, and soybean plants. (A) Relative expression level of *GmbZIP60* in Col-0, *OE-16*, and *OE-44* in transgenic *Arabidopsis*. (B) Relative expression level of *GmbZIP60* in William82, *OE-12*, and *OE-44* in transgenic soybean. (C) Relative expression level of *GmbZIP60* in ZH11, *OE-2*, and *OE-5* in transgenic rice. Errors bars indicate ± SD of three biological replicates. Asterisks indicate significant differences for the indicated comparisons based on a student's t-test (** $p < 0.01$; $0.01 <$ * $p < 0.05$).

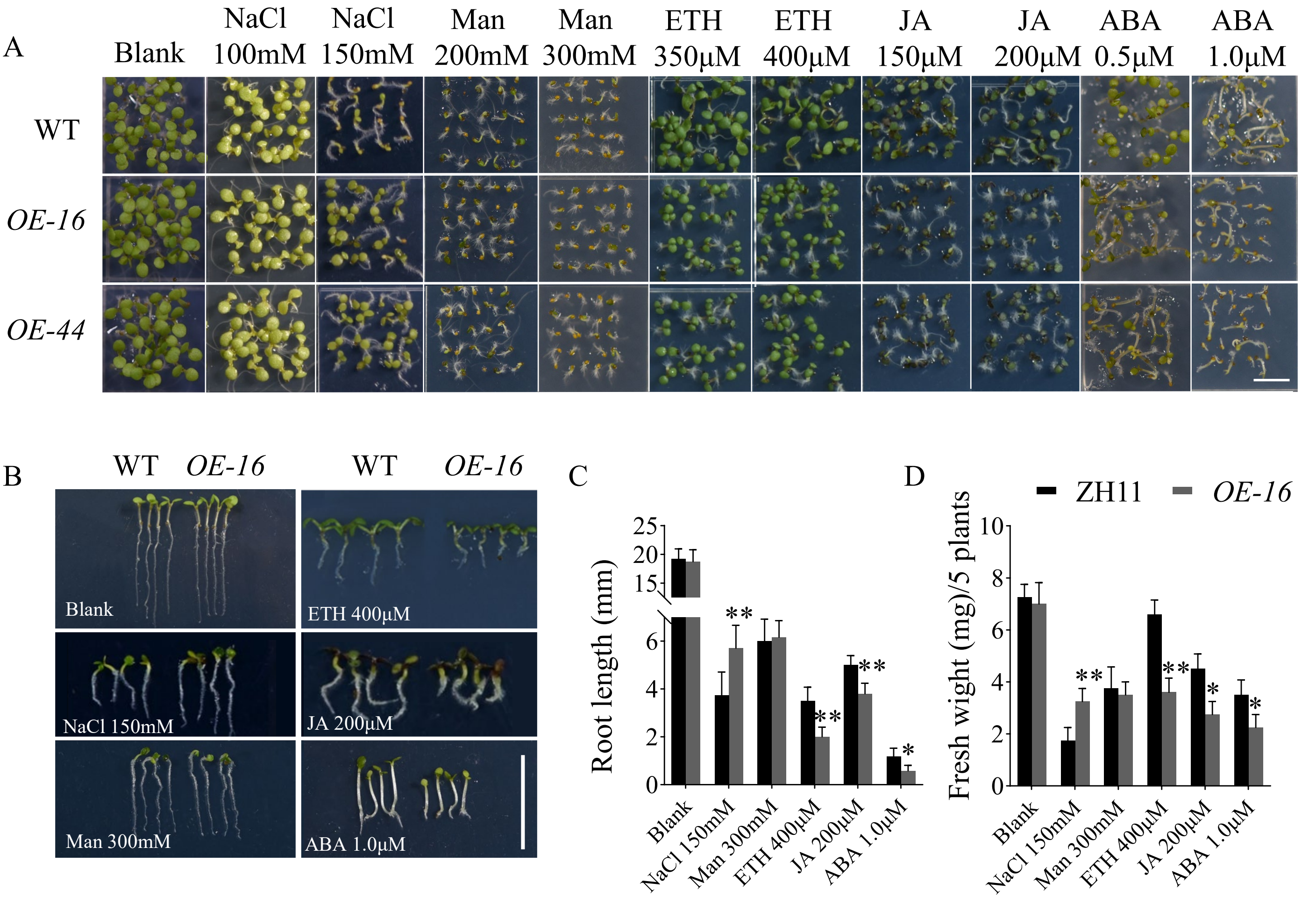

Supplemental Figure S3. (A) Phenotypic analysis of *OE-GmbZIP60* transgenic *Arabidopsis* plants in response to salt, plant hormones treatment in *Arabidopsis*. (B) All the seeds were germinated on the 1/2 Murashige and Skoog Medium (MS) medium under normal conditions (Blank) or supplemented with NaCl (100 mM and 150 mM), ETH (350μm and 400μm), and JA (150μm and 200μm) for 1 week (Scale bar, 1 cm). (C) Calculation of the seedlings' root length. (D) Calculation of the seedlings' fresh weights. *GmbZIP60* overexpression transgenic *Arabidopsis* plants (*OX-GmbZIP60-1* and *OX-GmbZIP60-2*, two independent transgenic lines). Methyl jasmonic acid (MeJA) and Ethylene (ETH). Errors bars indicate ± SD of three biological replicates. Asterisks indicate significant differences for the indicated comparisons based on a student's t-test (** $p < 0.01$; $0.01 <$ * $p < 0.05$).

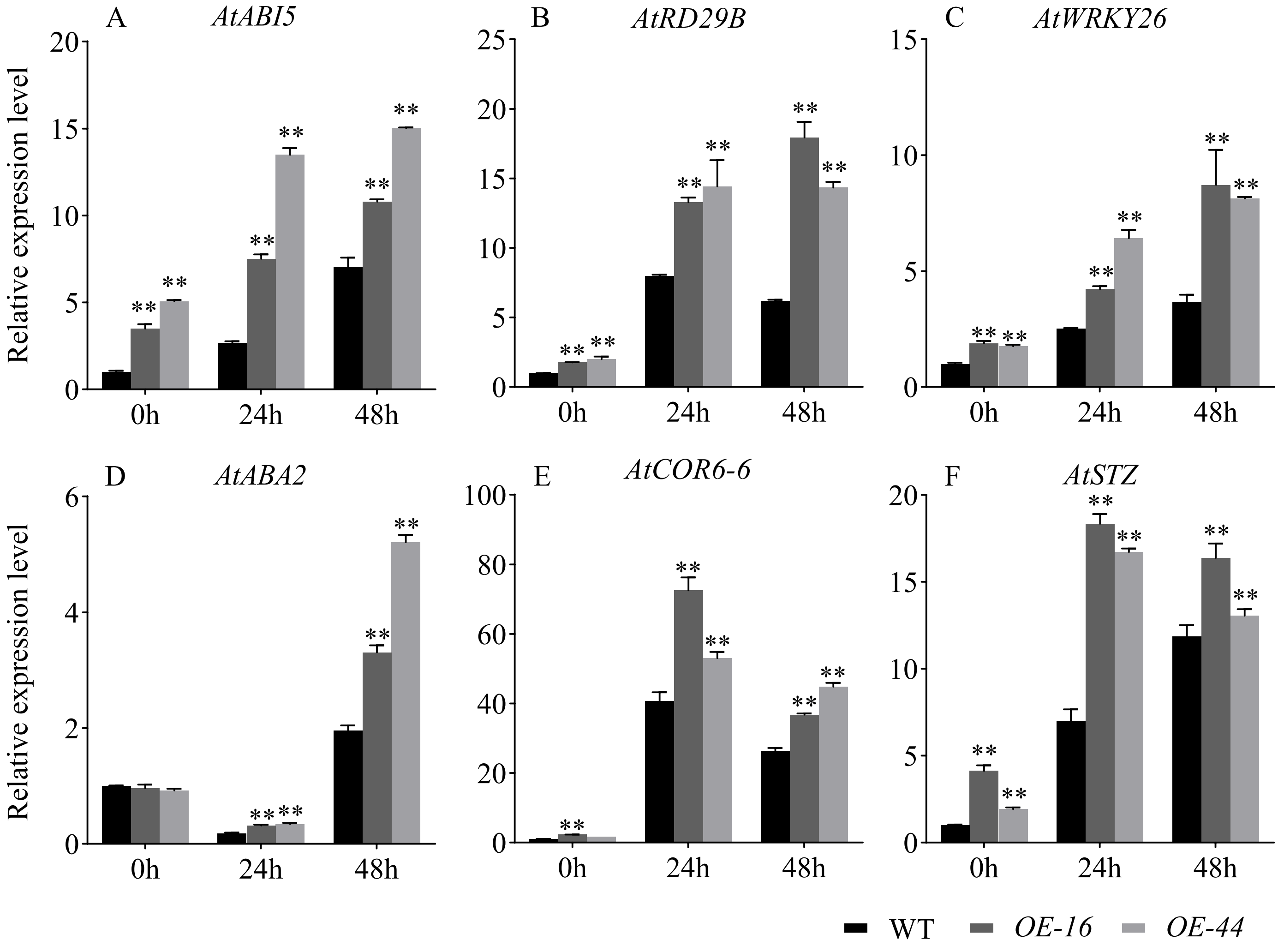

Supplemental Figure S4. The expression of abiotic stress-related genes in the WT and *OE-GmbZIP60* transgenic *Arabidopsis* plants in response to salt (A-F). Errors bars indicate ± SD of three biological replicates. Asterisks indicate significant differences for the indicated comparisons based on a student's t-test (** $p < 0.01$; $0.01 < * p < 0.05$).
